# Pregabalin Failed to Prevent Dry Eye Symptoms after Laser-Assisted in Situ Keratomileusis (LASIK) in a Randomized Pilot Study

**DOI:** 10.3390/jcm8091355

**Published:** 2019-09-01

**Authors:** Anat Galor, Sneh Patel, Leslie R. Small, Adriana Rodriguez, Michael J. Venincasa, Stephen E. Valido, William Feuer, Roy C. Levitt, Constantine D. Sarantopoulos, Elizabeth R. Felix

**Affiliations:** 1 Department of Ophthalmology, Miami Veterans Administration Medical Center, Miami, FL 33136, USA; 2 Bascom Palmer Eye Institute, University of Miami, Miami, FL 33136, USA; 3 Department of Anesthesiology, Perioperative Medicine and Pain Management, University of Miami, Miami, FL 33136, USA; 4 John P. Hussman Institute for Human Genomics, University of Miami Miller School of Medicine, Miami, FL 33136, USA; 5 John T. Macdonald Foundation Department of Human Genetics, University of Miami Miller School of Medicine, Miami, FL 33136, USA; 6 Department of Physical Medicine and Rehabilitation, University of Miami, Miami, FL 33136, USA; 7 Research Service, Miami Veterans Administration Medical Center, Miami, FL 33136, USA

**Keywords:** dry eye symptoms, dry eye, ocular pain, pregabalin, refractive surgery, LASIK

## Abstract

Purpose: Perioperative pregabalin administration has been found to reduce the risk of persistent pain after a variety of surgical procedures. However, this approach has not been tested in relation to eye surgery. As such, the purpose of this study was to evaluate whether perioperative pregabalin can reduce the presence of dry eye (DE) symptoms, including eye pain, six months after laser-assisted in situ keratomileusis (LASIK). Methods: Prospective, masked, randomized single-center pilot study. Patients were treated with either pregabalin (oral solution of pregabalin 150 mg twice daily, first dose prior to surgery, continued for a total of 28 doses over 14 days) or placebo solution. The primary outcome was dry eye symptoms as measured by the Dry Eye Questionnaire 5 (DEQ-5). Secondary outcome measures included pain-related eye symptoms. Results: In total, 43 individuals were enrolled in the study and randomized to pregabalin (*n* = 21) or placebo (*n* = 22). Of those, 42 individuals completed the final visit after six months of follow-up. Some differences were noted between the two groups at baseline, including a higher frequency of females in the pregabalin group. At 6-months, there were no significant differences in the percentage of patients with DE symptoms (DEQ5 ≥ 6, 57% vs. 33%, *p* = 0.14), DE symptom severity (DEQ5, 6.6 ± 5.0 vs. 4.5 ± 4.2, *p* = 0.14), ocular pain intensity (numerical rating scale, 1.10 ± 1.48 vs. 0.38 ± 0.97, *p* = 0.08), or neuropathic pain complaints (Neuropathic Pain Symptom Inventory-Eye, 2.81 ± 4.07 vs. 3.14 ± 5.85, *p* = 0.83) between the pregabalin and control groups. Ocular signs were likewise similar between the groups, and of note, did not correlate with DE symptoms. The strongest predictor of DE symptoms six months post-surgery was the presence of DE symptoms prior to surgery. Conclusions: Perioperative pregabalin did not reduce the frequency or severity of DE symptoms at a six month follow-up after LASIK in this small pilot study.

## 1. Introduction

Laser in-situ keratomileusis (LASIK) is a commonly performed surgical procedure used to correct the refractive error, and while visual outcomes after surgery tend to be excellent, a potential side effect of the procedure is the onset of persistent dry eye (DE) symptoms. The Patient Reported Outcomes with LASIK (PROWL) study reported that up to 27% of individuals reported mild or greater DE symptoms six months after LASIK (58 of 216), with new DE symptoms reported in 20% of patients (23/118) [1]. Such figures, within the published range of 20–55% occurrence, have been reported in several retrospective studies [2,3,4,5,6,7,8]. Post-LASIK DE symptoms were initially attributed to ocular surface dryness; however, recent evidence suggests that these symptoms may result from corneal nerve damage induced by LASIK; in fact, a recent review highlighted that corneal nerve density might not return to baseline levels, even years after surgery [9].

Further support for the role of nerve dysfunction in post-LASIK DE comes from the use of neuropathic pain descriptors in many individuals. The concept of neuropathic pain as applied to the eye represents a manifestation of pathological neuroplasticity of the trigeminal somatosensory system and higher pathways associated with the spontaneous firing of primary afferents (peripheral sensitization), trigeminal, and/or higher sensory neurons (central sensitization) [10]. Specific DE-related complaints in post-LASIK patients include spontaneous burning pain, pain evoked by wind or light (i.e., hyperalgesia and allodynia), and discordance between ocular pain symptoms and signs of corneal disease [11]. While most individuals experience only mild symptoms, some individuals experience more severe symptoms that can negatively impact the quality of life [1]. Even further, some patients experience such severe pain that they must pursue invasive procedures, including trigeminal nerve stimulation or intrathecal drug delivery, to obtain relief from pain [12]. 

No current strategies exist to prevent the development of DE symptoms post-LASIK. However, given that DE symptoms, due to LASIK resemble persistent postoperative pain (PPOP), which is known to occur after other surgical procedures, strategies used to prevent PPOP may also be useful to patients undergoing LASIK [13]. Pre-emptive analgesia is one such strategy, which entails anti-nociceptive treatment around the time of surgery to prevent the onset of post-surgical chronic pain [14]. Gabapentin and pregabalin (α2δ ligands) are the most commonly used agents in this regard, and have been found to reduce the incidence and severity of persistent pain after many other surgeries, including mastectomy, thoracotomy, and hernia repair [14]. However, it is not known whether α2δ ligands can decrease the presence of painful DE symptoms after LASIK. Based on this rationale, we designed a pilot study to assess whether preemptive analgesia with pregabalin could reduce the incidence of post-LASIK painful DE symptoms.

## 2. Methods

This randomized pilot study was registered with clinicaltrials.gov, with registry information as follows—NCT02701764, registered March 8, 2016, completed December 2018. 

### 2.1. Study Population

The institutional review board (IRB) of the University of Miami approved this prospective study, the methods adhered to the tenets of the Declaration of Helsinki, and all patients signed an informed consent form prior to participation. Inclusion criteria consisted of subjects who elected to undergo LASIK at the Bascom Palmer Eye Institute and who were on stable ocular and systemic medication for at least the past three months. Exclusion criteria included: Pregnancy at time of recruitment, use of gabapentin, pregabalin, antiepileptics, duloxetine, venlafaxine or tri-cyclic antidepressants; chronic use (or within one month prior to surgery) of corticosteroids; history of corneal disease; prior corneal incisions; use of topical medications other than for DE; and the presence of systemic diseases that could confound DE (e.g., human immunodeficiency virus, sarcoidosis, graft-versus-host disease, or collagen vascular disease).

### 2.2. Intervention

The study was a single-center, randomized, double-masked, and placebo-controlled. Subjects were randomized in a 1:1 ratio into pregabalin (150 mg (7.5 cc) P.O. B.I.D. starting one day before surgery and continued for 28 doses for 14 days total) or placebo. A variable (*n* = 2 or *n* = 4) blocked randomization list for treatment assignments was constructed (W.F.) to ensure that treatment assignments were balanced after, at most four patients. Each subject received 28 similarly labeled and colored syringes containing either 7.5 mL of pregabalin (20 mg/mL solution, equivalent of 150 mg) or placebo (7.5 mL). The placebo was created by adding 14 mL of Strawberry Flavor (Letco #685093) to 480 mL of non-flavored oral syrup (Letco #695076). Each patient was also treated with a corticosteroid (prednisolone acetate 1%) and antibiotic (moxifloxacin) Q.I.D. for one week after the procedure. 

### 2.3. Study Variables

#### 2.3.1. DE Symptoms

Two validated DE questionnaires, the Dry Eye Questionnaire 5 (DEQ5) [15] and Ocular Surface Disease Index (OSDI) [16] were administered. These questionnaires are commonly used to evaluate different aspects of DE symptoms and their impact, and have been validated in several studies [15,16,17].

#### 2.3.2. Ocular Pain

Ocular pain: Three ocular pain questionnaires were administered, each targeting different aspects of pain: (a) Numerical rating scale (NRS) of ocular pain intensity (range 0–10; “no pain” to “most intense pain imaginable”) over a one week recall period (average eye pain during the past week, and worst eye pain during the past week) [18,19] (b) short form McGill Pain Questionnaire (sf-MPQ) [20], consisting of 15 words, including sensory and affective descriptors of pain over a one week recall (range 0–45); (c) the Neuropathic Pain Symptom Inventory modified for the eye (NPSI-Eye) [21], evaluating symptoms of neuropathic pain over a 24 h recall (range 0–100). The NPSI-Eye is a modified version of the original NPSI [22] in which questions 8, 9, and 10 were altered to be specific to ocular allodynia and hyperalgesia (ocular pain evoked or worsened by (1) light, (2) wind, and (3) heat or cold). The NRS is the primary outcome measure suggested for use in clinical trials of pain [23,24] and has shown outstanding validity and reliability across a number of patient groups [25,26,27]. Likewise, the sf-MPQ has also demonstrated excellent psychometric properties [28,29]. The original NPSI has been validated as a reliable measure for severity of neuropathic pain (citations) [30,31,32], and the NPSI-Eye has been validated specifically for indicating the severity of neuropathic characteristics of eye pain [16].

#### 2.3.3. Visual Acuity

Snellen visual acuity testing. Uncorrected and best-corrected visual acuity was obtained at each visit, and recorded along with the manifest refraction.

#### 2.3.4. Ocular Surface Testing

Ocular surface testing in order included: (a) Inflammadry testing (Quidel, San Diego, CA, USA), tear film break up time (TBUT) (average of three measures, each eye), conjunctival and corneal staining assessed using the National Eye Institute (NEI) scoring scale, eyelid assessment. For example, the degree of anterior blepharitis and eyelid vascularity were scored on a scale of 0 to 3 (0 = none; 1 = mild; 2 = moderate; 3 = severe), as was the degree of inferior eyelid meibomian orifice plugging (0 = none; 1 = less than 1/3 lid involvement; 2 = between 1/3 and 2/3 lid involvement; 3 greater than 2/3 lid involvement). The presence of fibrosis, papillary, or follicular conjunctival changes was determined as either absent or present. Meibomian gland drop-out was assessed via meibography and graded to the Meiboscale. Schirmer strips were used to measure tear production; strips were placed in the outer 1/3 of the lower conjunctivae and the length of wetting after five minutes recorded [33]. Meibum quality was then rated as follows: 0 = clear; 1 = cloudy; 2 = granular; 3 = toothpaste; 4 = none extracted [34].

#### 2.3.5. Assessed Co-Morbidities

Many factors, besides pregabalin use, can affect DE symptoms, including medications and co-morbidities. As such, we collected information on variables of interest, including: (a) Demographics; (b) medications; (c) co-morbidities (diabetes, hypertension); (d) non-ocular pain via a pain history questionnaire; (e) depression and anxiety via the Symptoms Checklist (SCL)-90; and (f) surgical factors (treatment parameters, flap thickness).

#### 2.3.6. Side Effects

All individuals filled out a standardized adverse events questionnaire via telephone interview two weeks after surgery.

#### 2.3.7. Main Outcome Measures

The main outcome measure was the percentage of patients that showed clinically significant levels of DE symptoms at six months, defined as having a DEQ5 score ≥ 6. Secondary outcome measures included the intensity of DE symptoms (DEQ5, OSDI), overall eye pain (NRS), and specific eye pain descriptors (sf-MPQ, NPSI-Eye). All listed measures were recorded three times in total, once prior to surgery and twice at three and six months after surgery. The rationale behind using symptom-based outcomes comes from recommendations by the IMMPACT (Initiative on Methods, Measurement, and Pain Assessment in Clinical Trials) group [23], the NIH task force report on chronic low back pain [35], and several precedent initiatives [36,37,38,39]. The IMMPACT group, in particular, outlined that pain dimensions (e.g., intensity, location, descriptors, qualities) should be assessed as core outcome measures for clinical trials, for example, a 0–10 NRS of pain intensity, in order to facilitate consistency among studies [23,24]. Given our focus on both DE symptoms and eye pain, we choose two validated DE questionnaires (DEQ5 [15] and OSDI [16]) and one validated eye pain questionnaires (NPSI-Eye) [22], in addition to using the 0–10 NRS pain intensity scale, recommended as a primary endpoint for pain clinical trials by the IMMPACT group.

### 2.4. Statistical Analysis

All statistical analyses were performed using SPSS 22.0 [40]. The principal analysis was a frequency comparison of DE symptoms (DEQ5 score ≥ 6) between the two groups at six months using a Chi square methodology. This analysis was performed on an intent-to-treat basis (ITT). Secondary analyses included mean comparisons of DE symptoms, ocular pain, and DE signs between the groups. Multivariable analyses using forward step-wise logistic and linear regression modeling were performed to consider the contribution of demographics, baseline examination findings, co-morbidities, and LASIK treatment parameters on DE symptoms. All statistical tests were two-sided tests and conducted at a nominal 5% level of significance.

### 2.5. Power Calculation

For this pilot study, our intent was to assess the potential effect of pregabalin on DE symptoms after LASIK, while limiting resources expended and time to study completion. Assuming 55% persistent DE in the placebo group reduced to 20% in the pregabalin treated group and analysis with the Chi-square test, a sample size of 22 patients per group gives us approximately 70% power to detect a difference, with an alpha error of 0.05 for this study. For this study, a loss to follow up rate of 10% was deemed acceptable. We acknowledge that these estimates are on the upper end of the expected effect and performed this pilot study to generate estimates on the potential benefit of this approach that can be used to develop larger, well-powered clinical studies.

## 3. Results

### 3.1. Study Population

**Figure 1:**
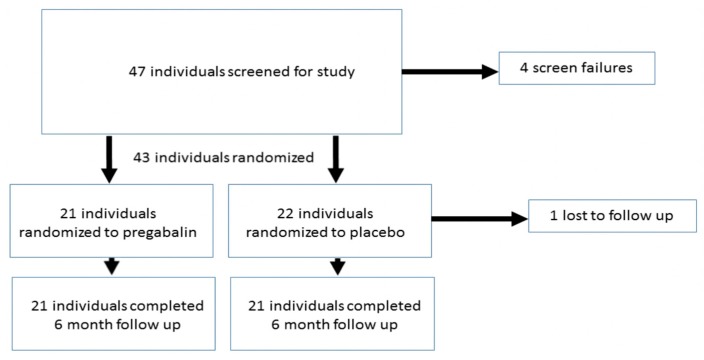
Patient Recruitment and Randomization Flow Chart.

In total, 43 individuals were enrolled and randomized into the pregabalin (*n* = 21) or placebo (*n* = 22) groups. Of these, 42 patients completed the final visit at six months (Figure 1).

Our cohort was young (mean age 35 ± 9.9 years) and healthy—no patient had diabetes, one patient had hypertension, one patient had sleep apnea, one patient had arthritis, and one patient had thyroid disease. Some differences were noted between our two groups at baseline, including a higher percentage of females and higher intensity of eye pain (measured via sf-MPQ) in the pregabalin group. Overall, 44% of the population had DE symptoms at baseline (DEQ5 score ≥ 6), but the majority of these individuals had only mild symptoms (Table 1).

### 3.2. Ocular Symptoms Three Months after LASIK

Overall, the percentage of participants showing mild or greater DE symptoms was higher at three months than when measured at baseline (56% of population showed DEQ5 ≥ 6), but on average symptoms were in the mild range for both groups. The number of patients using artificial tears was also higher post-LASIK when compared to use prior to LASIK, but usage rates were similar across both groups (55% vs. 46%%, *p* = 0.54). A greater percentage of individuals that were randomized to the pregabalin group showed significant DE symptoms at three months compared to the placebo group (67% vs. 46%; *p* = 0.16), although the difference was not significant (Table 2).

### 3.3. Ocular Symptoms and Signs at Six Months after LASIK

**Figure 2:**
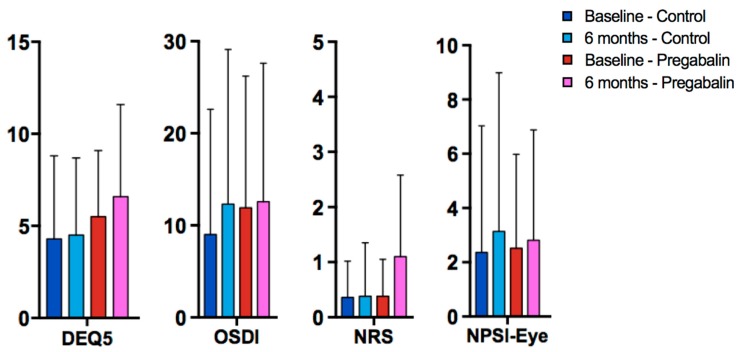
Comparison of Ocular Symptom Scores at Baseline and 6-Months. DEQ5 = Dry Eye Questionnaire 5, OSDI = Ocular Surface Disease Index, NRS = Numerical Rating Scale for ocular pain averaged over one week recall, NPSI-Eye = Neuropathic Pain Symptom Inventory modified for an eye.

The frequency of mild or greater DE symptoms after six months returned to levels similar to those observed at baseline (45% of the population had DEQ5 ≥ 6) (Table 3). Also, as seen at 3-month follow-up, more individuals randomized to the pregabalin group presented with DE symptoms than those patients randomized to placebo (57% vs. 33%; *p* = 0.12), but this difference was not statistically significant (Figure 2). The difference between the groups was 24%, and the 95% confidence interval ranged from −53% to +5%. Ocular signs were similar between the two groups at six months follow-up. More individuals in the pregabalin group used artificial tears at six months, but the difference was again not statistically significant (57% vs. 48%, *p* = 0.54). In total, three patients (two in the pregabalin group and one in control) had punctal plugs placed by six months.

To examine the validity of our outcome measures, we ran Pearson correlations to assess the strength of association between measures of eye pain intensity (Table 4). The three pain indices (NRS, NPSI-Eye, sf-MPQ) were strongly correlated with one another (average *r* = 0.66). The two overall symptom indices (DEQ5, OSDI) were weakly correlated (*r* = 0.34), although this is not surprising given that the two questionnaires measure different aspects of DE. The DEQ5 focuses on symptoms (dryness, discomfort, tearing), while the OSDI also incorporates DE triggers and impact on everyday activities.

### 3.4. Factors Predictive of DE Symptom Frequency and Severity Six Months after Surgery

Multivariable linear regression modeling was used to determine which baseline factors in Table 1 (demographics, comorbidities, medications, LASIK treatment profile) influenced the presence of ocular symptoms at six months, while adjusting for treatment allocation (pregabalin vs. placebo) and ocular surface signs at six months (Table 5). The most robust predictor of DE symptoms at six months was having DE symptoms at baseline. For instance, baseline DEQ5 score and age predicted 44% of variability in DEQ5 at six months (standardized beta = 0.74, *p* < 0.0005 for DEQ5 and 0.13, *p* = 0.03 for age). Similarly, baseline OSDI score predicted 11% of variability in OSDI score at six months (standardized beta = 0.33, *p* = 0.04). On the other hand, ocular pain at six months was influenced more heavily by mental health indices. For example, average ocular pain over one week recall (NRS) was most influenced by baseline anxiety (via Scl-90, standardized beta = 0.61, *p* < 0.0005), sf-MPQ sensory score was most influenced by baseline depression (via Scl-90, standardized beta = 0.73, *p* < 0.0005), and total NPSI-Eye score was influenced by both baseline depression (via Scl-90, standardized beta = 0.50, *p* < 0.0005) and baseline eye pain (standardized beta = 0.45, *p* < 0.0005), *R* = 0.79.

### 3.5. Visual Outcomes and Side Effects

Visual outcomes were excellent, with 86% of individuals in both groups achieving the uncorrected vision of 20/25 or better in both eyes. All but one patient (who developed an epitheliopathy in the setting of pre-existing map dot fingerprint dystrophy) maintained the best-corrected vision of 20/20 in both eyes at six months. Subjects in the pregabalin group reported a higher frequency of side effects when compared to the control group, namely tiredness and dizziness, as assessed two weeks after surgery (Table 6).

## 4. Discussion

In our pilot study, we found that perioperative pregabalin did not reduce the presence of DE symptoms six months after LASIK. This included overall symptoms of DE, assessed using standardized questionnaires like the DEQ5 and OSDI, and symptoms related to pain, assessed via standardized pain questionnaires applied to eye pain e.g., NRS, NPSI-Eye, and sf-MPQ.

There are several explanations for our findings. First, it is possible that α2δ ligands are not the optimal agents to prevent mechanisms that lead to ocular symptoms. Other agents, such as anti-depressants (tricyclics and serotonin and norepinephrine reuptake inhibitors) have been used as preventative agents in PPOP, and may be better suited for controlling eye pain. Furthermore, combination therapies, such as using an α2δ ligand and an analgesic antidepressant (e.g., nortriptyline), might have been more beneficial via targeting of multiple mechanisms contributing to the transition from acute to chronic ocular pain. Combined therapy with gabapentin and nortriptyline, for example, has been shown to suppress neuropathic pain wherein individual components may be ineffective [41]. Second, it is possible that our dosing strategy was not optimal. In the literature, dosing strategies vary tremendously and range from exclusively high dose preoperative administration, to a low preoperative dose followed by an extended taper [14,42]. Third, it is possible that our study population was not optimal. Additionally, the overall severity of DE symptoms and ocular pain in our population at six months were low, and second, despite randomization, our groups were not matched at baseline with respect to gender. However, noted baseline differences, specifically female gender, did not predict ocular symptoms at six months, although female gender has been identified as a risk factor for PPOP in other studies [42,43,44]. Given that baseline ocular symptoms and mental health indices (e.g., depression, anxiety) were most influential in predicting symptoms at six months, restricting our population to those with these risk factors may have been a more optimal strategy in testing our hypothesis.

Finally, it is likely that a single systemically administered analgesic agent, such as pregabalin, although adequate to reduce symptoms of established neuropathic pain, may not be adequate to suppress the entire afferent barrage of nociceptive signals or chemical mediators originating from the cornea and affecting the central nervous system leading to sensitization and chronicity of pain [45]. Although the concept of preemptive analgesia, as originally introduced by Woolf and Chong, promoted the preemptive use of analgesics to prevent central sensitization, subsequent studies produced contradictory results [46]. Therefore, what might be necessary to more effectively suppress peripheral and central sensitization and reduce the transition to chronicity is combined preventive application of multiple classes of analgesics from different routes, such as local anesthetics and multimodal systemic agents of different classes, extending from the preoperative phase to the postoperative period [46,47]. In fact, this is already utilized in LASIK in the form of topical anesthetics during surgery and topical corticosteroids in the postoperative period.

According to existing PPOP literature, the frequency of mild DE symptoms in this study (45% at six months) is higher than PPOP rates after dental implant surgery (8.5–36%) [48], as well as inguinal hernia repair (5–30%) [49], and is similar to PPOP rates post-thoracotomy (5–65%) [49] and breast surgery (20–50%) [49]. Fortunately, most individuals had mild symptoms, with only 12% of subjects reporting severe symptoms. In a similar manner, of the 14 subjects who reported eye pain over a one week recall, 12 patients rated it as mild (1–3) [50]. None of our patients had debilitating symptoms that necessitated escalation of therapy beyond that typically used to address DE (artificial tears, punctal plugs). Consistent with prior PPOP studies, pre-existing pain, anxiety, and depression were risk factors for persistent ocular symptoms six months after surgery [51].

Gabapentin and pregabalin have also been studied in the treatment of acute pain after photorefractive keratectomy (PRK). Two studies found no difference in subjective pain ratings immediately after PRK between gabapentin (300 mg three times daily) and oxycodone/acetaminophen (5 mg/325 mg) [52] or placebo [53]. Two studies, on the other hand, found that α2δ ligands reduced acute postoperative pain during the first 72 h after surgery [54,55]. Our study targeted a different aspect of pain, namely prevention of chronic ocular symptoms (dryness, pain), after LASIK.

The chief limitation of our study was its small sample size. However, given our confidence interval of −53% to 5% for the difference in frequency of DE symptoms between the groups, we have good cause to rule out a reduction greater than 5% in persistent DE symptoms in the pregabalin compared to control. This suggests that pregabalin does not have a clinically substantial benefit on the frequency of DE symptoms at the current dose and the current population studied. As such, future studies with a larger population of individuals at higher risk of DE symptoms after LASIK (those with pre-existing pain, depression, and/or anxiety) are needed to re-examine this question. Furthermore, our groups were not matched at baseline despite randomization. However, these baseline differences were not significant predictors of DE symptoms at six months, and as such, this occurrence does not seem to have confounded the study. Furthermore, we did not image cornea nerves, and thus, do not have data on differences in nerve regrowth between the groups after surgery. While many studies found that that α2δ ligands suppress ectopic discharge activity from injured nerve sites [56,57,58,59], few have studied how this blockade affects nerve healing. One study on sciatic crush injury in rats found that low dose pregabalin (30 mg/kg) over four weeks promoted nerve regeneration and functional recovery, while high doses (60 mg/kg) did not [59]. On the other hand, another study of pregabalin 10 mg/kg over three weeks did not find an effect on nerve regeneration [60]. A third study found that pregabalin-collagen therapy after injury aided in nerve regeneration by inhibiting IL-10 release, a pro-inflammatory cytokine that negatively impacts the healing processes [61]. Our current study does not provide information on the effects of pregabalin on corneal nerve regeneration and as such, future studies are needed that focus on these connections, such as with the use of confocal microscopy. Of note, we focused on LASIK in this study given an epidemiological link between LASIK and DE symptoms; however, newer surgical techniques, like small incision lenticule extraction (SMILE), are now available and may lower the overall incidence of post-refractive DE symptoms in the future [62].

## 5. Conclusions

To conclude, we found that perioperative pregabalin did not reduce the frequency or severity of ocular symptoms six months after LASIK. Future studies may need to reconsider medication choice, dosing strategy, and population when studying preventative strategies for post-LASIK DE.

## Figures and Tables

**Table 1:** Comparison of demographics, co-morbidities, DE metrics, and treatment parameters between the two groups at baseline.

	Pregabalin	Control	*p-*Value
Number	21	22	
Demographics			
Age, years, mean (SD)	37.8 (9.8)	33.0 (0.6)	0.12
Female, *n* (%)	14 (67%)	8 (36%)	0.047
Race, white, *n* (%)	18 (86%)	18 (82%)	0.92
Hispanic, *n* (%)	11 (52%)	11 (50%)	0.88
Surgical information, mean (SD)			
Spherical equivalent of treatment, D *	−3.48 (3.25)	−3.95 (1.79)	0.56
Flap depth, microns, mean (SD)	117 (21)	109 (30)	0.34
Eye co-morbidities, *n* (%)			
Self-reported eye allergies	1 (5%)	2 (9%)	1.0
Contact lens wear prior to surgery	16 (76%)	13 (59%)	0.23
Artificial tears use prior to surgery	4 (19%)	5 (23%)	1.0
Eye symptoms			
DEQ5, mean (SD), range 0–22	5.5 (3.6)	4.3 (4.5)	0.34
DEQ5, mild symptoms ≥6, *n* (%)	10 (48%)	9 (41%)	0.66
DEQ5, severe symptoms ≥12, *n* (%)	2 (10%)	2 (10%)	0.96
OSDI, mean (SD), range 0-100	11.9 (14.3)	9.0 (13.6)	0.50
Ocular pain, worst over one week recall, mean (SD), range 0–10	0.48 (0.75)	0.64 (0.75)	0.61
Ocular pain, average over one week recall, mean (SD), range 0–10	0.38 (0.67)	0.36 (0.66)	0.93
Sf-MPQ sensory, mean (SD), range 0–33	0.90 (1.84)	0.09 (0.29)	0.06
Sf-MPQ affective, mean (SD), range 0–12	0.43 (0.68)	0.23 (0.61)	0.31
Intensity of burning over one day recall, mean (SD), range 0–10	0.10 (0.30)	0.27 (0.63)	0.25
Intensity of wind sensitivity one day recall, mean (SD), range 0–10	0.48 (0.68)	0.36 (1.09)	0.69
Intensity of light sensitivity one day recall, mean (SD), range 0–10	0.86 (1.42)	0.68 (1.81)	0.73
NPSI-Eye total, mean (SD), range 0–100	2.52 (3.46)	2.36 (4.67)	0.90
Eye signs *			
Any eyelid laxity, *n* (%)	3 (14%)	2 (9%)	0.66
Inflammadry score ≥2, *n* (%)	4 (19%)	3 (14%)	0.63
Inflammadry positive, *n* (%)	11 (52%)	15 (68%)	0.07
TBUT, seconds, mean (SD)	10.9 (7.3)	9.1 (6.2)	0.67
Conjunctivochalasis, *n* (%)	12 (57%)	16 (73%)	0.28
Corneal staining, mean (SD)	2.2 (2.5)	2.6 (2.7)	0.66
Anterior blepharitis, mean (SD)	0.95 (0.80)	1.00 (0.82)	0.85
Eyelid vascularity, mean (SD)	1.00 (0.95)	0.77 (0.81)	0.40
Meibomian plugging, mean (SD)	1.33 (0.91)	1.41 (0.91)	0.79
Meibomian gland drop, mean (SD)	1.24 (1.04)	0.91 (0.75)	0.24
Schirmers test, mm wetting, mean (SD)	12.8 (7.4)	17.0 (9.0)	0.10
Meibomian quality, mean (SD)	1.95 (1.32)	1.59 (1.18)	0.35
Conjunctival papillae, ≥mild, *n* (%)	15 (71%)	14 (64%)	0.86
Co-morbidities			
Average daily screen time, hours, mean (SD)	8.7 (4.1)	7.9 (3.0)	0.44
Depression via Scl-90, mean (SD), range 0–4	0.32 (0.33)	0.28 (0.64)	0.82
Anxiety via Scl-90, mean (SD), range 0–4	0.24 (0.29)	0.13 (0.24)	0.16
Carpet at home, *n* (%)	5 (24%)	4 (18%)	0.65
Non-ocular allergies, *n* (%)	5 (24%)	5 (23%)	0.93
Chronic pain in any area, *n* (%)	5 (24%)	4 (18%)	0.72
Headache (including migraine), *n* (%)	3 (14%)	2 (9%)	0.66
No of chronic pain conditions (headache, low back pain, sciatica, irritable bowel), mean (SD)	0.52 (1.33)	0.23 (0.61)	0.35
Average non-ocular pain intensity, one week recall, mean (SD)	0.71 (1.62)	0.50 (1.19)	0.62

SD = Standard deviation, D = diopters, DEQ5 = Dry Eye Questionnaire 5, OSDI = Ocular Surface Disease Index, sf-MPQ = short form McGill Pain Questionnaire, NPSI-Eye = Neuropathic Pain Symptom Inventory modified for the eye, TBUT = tear break up time, SCL = Symptom Checklist; * Unless indicated, value from more severely affected eye used in analysis.

**Table 2:** Ocular symptoms at three months post-LASIK, by treatment assignment.

	Pregabalin	Control	*p*-Value
Number	21	22	
Eye symptoms			
DEQ5, mean (SD), range 0–22	6.6 (3.9)	4.7 (4.4)	0.14
DEQ5, mild symptoms ≥6, *n* (%)	14 (67%)	10 (46%)	0.16
DEQ5, severe symptoms ≥12, *n* (%)	1 (5%)	2 (9%)	1.00
Change in DEQ5 from baseline, mean (SD)	1.1 (3.9)	0.4 (4.0)	0.54
OSDI, mean (SD), range 0–100	11.9 (11.5)	11.0 (16.6)	0.84
Change in OSDI from baseline, mean (SD)	−0.01 (15.6)	2.0 (15.1)	0.67
Ocular pain, worst over one week recall, mean (SD), range 0–10	1.25 (1.52)	0.50 (1.01)	0.07
Ocular pain, average over one week recall, mean (SD), range 0–10	0.85 (0.27)	0.27 (0.55)	0.07
Sf-MPQ sensory, mean (SD), range 0–33	0.65 (1.23)	0.36 (0.66)	0.35
Sf-MPQ affective, mean (SD), range 0–12	0.30 (0.66)	0.18 (0.39)	0.48
Intensity of burning over one day recall, mean (SD), range 0–10	0.15 (0.67)	0.64 (1.76)	0.24
Intensity of wind sensitivity one day recall, mean (SD), range 0–10	0.70 (1.56)	0.36 (0.95)	0.40
Intensity of light sensitivity one day recall, mean (SD), range 0–10	0.90 (1.83)	0.64 (1.62)	0.62
NPSI-Eye total, mean (SD), range 0–100	2.70 (4.55)	1.86 (4.07)	0.53

SD = Standard deviation, DEQ5 = Dry Eye Questionnaire 5, OSDI = Ocular Surface Disease Index, sf-MPQ = short form McGill Pain Questionnaire, NPSI-Eye = Neuropathic Pain Symptom Inventory for the eye.

**Table 3:** Ocular symptoms and signs at six months post-LASIK, by treatment assignment.

	Pregabalin	Control	*p*-Value
Number (n)	21	21	
Eye symptoms			
DEQ5, mean (SD), range 0–22	6.6 (5.0)	4.5 (4.2)	0.14
DEQ5, mild symptoms ≥6, *n* (%)	12 (57%)	7 (33%)	0.12
DEQ5, severe symptoms ≥12, *n* (%)	4 (19%)	1 (5%)	0.34
Change in DEQ5 from baseline, mean (SD)	1.1 (3.9)	0.1 (3.6)	0.42
OSDI, mean (SD), range 0–100	12.6 (15.0)	12.3 (16.8)	0.96
Change in OSDI from baseline, mean (SD)	-0.30 (14.4)	2.9 (15.9)	0.51
Ocular pain, worst over one week recall, mean (SD), range 0–10	1.38 (1.75)	0.76 (1.38)	0.21
Ocular pain, average over one week recall, mean (SD), range 0–10	1.10 (1.48)	0.38 (0.97)	0.08
Intensity of burning over one day recall, mean (SD), range 0–10	0.43 (0.87)	0.62 (1.28)	0.58
Any burning pain, *n* (%)	5 (24%)	5 (24%)	1.00
Intensity of wind sensitivity one day recall, mean (SD), range 0–10	1.00 (1.95)	0.57 (0.93)	0.37
Any sensitivity to wind, *n* (%)	7 (33%)	7 (33%)	1.00
Intensity of light sensitivity one day recall, mean (SD), range 0–10	0.48 (1.25)	0.86 (1.49)	0.38
Any sensitivity to light, *n* (%)	4 (19%)	8 (38%)	0.17
NPSI-Eye total, mean (SD), range 0–100	2.81 (4.07)	3.14 (5.85)	0.83
Eye signs *			
Inflammadry score ≥2, *n* (%)	2 (10%)	4 (20%)	0.66
Inflammadry positive, *n* (%)	10 (50%)	7 (35%)	0.34
TBUT, seconds, mean (SD)	8.35 (2.46)	9.05 (5.93)	0.62
Corneal staining, mean (SD)	2.43 (2.58)	2.05 (1.94)	0.59
Schirmer score, mm wetting, mean (SD)	15.45 (8.17)	15.05 (8.64)	0.88

SD = Standard deviation, DEQ5 = Dry Eye Questionnaire 5, OSDI = Ocular Surface Disease Index, sf-MPQ = short form McGill Pain Questionnaire, NPSI-Eye = Neuropathic Pain Symptom Inventory for the eye; * Unless indicated, value from more severely affected eye used in analysis.

**Table 4:** Relationships Between Ocular Symptom Score Questionnaires.

Pearson r	OSDI	NRS	sf-MPQ	NPSI-Eye	DEQ5
OSDI	*r* = 1	*r* = 0.12 *p* = 0.45	*r* = 0.26 *p* = 0.11	*r* = 0.20 *p* = 0.22	*r* = 0.34 *p* = 0.03
NRS		*r* = 1	*r* = 0.56 *p* < 0.0005	*r* = 0.68 *p* < 0.0005	*r* = 0.38 *p* = 0.01
sf-MPQ			r = 1	*r* = 0.75 *p* < 0.0005	*r* = 0.20 *p* = 0.22
NPSI-Eye				*r* = 1	*r* = 0.28 *p* = 0.08
DEQ5					*r* = 1

OSDI = Ocular Surface Disease Index; NRS = Numerical Rating System for average ocular pain over one week recall; sf-MPQ = Short Form McGill Pain Questionnaire; NPSI-Eye = Neuropathic Pain Symptom Inventory Modified for the eye; DEQ5 = Dry Eye Questionnaire 5.

**Table 5:** Multivariable Analyses on Factors Predictive of DE Symptom Frequency and Severity.

Model Outcome	Predictor Variable(s)	β	*p*-Value
DEQ5 at six months	DEQ5 (Baseline)	0.74	<0.0005
	Age	0.13	0.03
OSDI at six months	OSDI (Baseline)	0.33	0.04
NRS at six months	Scl-90 Anxiety (Baseline)	0.61	<0.0005
sf-MPQ at six months	Scl-90 Depression (Baseline)	0.73	<0.0005
NPSI-Eye at six months	Scl-90 Depression (Baseline)	0.50	<0.0005
	NRS (Baseline)	0.45	<0.0005

DEQ5 = Dry Eye Questionnaire 5; OSDI = Ocular Surface Disease Index; NRS = Numerical Rating System for average ocular pain over one week recall; sf-MPQ = Short Form McGill Pain Questionnaire; NPSI-Eye = Neuropathic Pain Symptom Inventory Modified for the eye; Scl-90 = Symptom Checklist 90.

**Table 6:** Side effects reported, by treatment assignment.

	Pregabalin	Control	*p*-Value
Any side effects, *n* (%)	13 (62%)	10 (46%)	0.28
Tiredness	8 (38%)	2 (9%)	0.03
Dizziness	6 (29%)	1 (5%)	0.05
Headache	3 (14%)	3 (14%)	1.0
Nausea	1 (5%)	2 (9%)	1.0
Dry mouth	3 (14%)	0 (0%)	0.11
Constipation	3 (14%)	1 (5%)	0.35
Bloating	3 (14%)	4 (18%)	1.0
High or elevated mood	4 (19%)	1 (5%)	0.19
